# Ethyl 6-(4-chloro­phen­yl)-4-(4-methoxy­phen­yl)-2-oxocyclo­hex-3-ene-1-carboxyl­ate

**DOI:** 10.1107/S1600536809016237

**Published:** 2009-05-07

**Authors:** Hoong-Kun Fun, Samuel Robinson Jebas, K. S. Girish, Balakrishna Kalluraya

**Affiliations:** a X-ray Crystallography Unit, School of Physics, Universiti Sains Malaysia, 11800 USM, Penang, Malaysia; b Department of Studies in Chemistry, Mangalore University, Mangalagangotri, Mangalore 574 199, India

## Abstract

In the title compound, C_22_H_21_ClO_4_, the cyclo­hex-3-ene unit adopts an envelope conformation in both independent mol­ecules comprising the asymmetric unit. The two benzene rings are inclined to each other at a dihedral angle of 82.03 (5)° [86.37 (5)°]. In the crystal, the molecules interact *via* C—H⋯O, C—H⋯Cl and C—H⋯π interactions.

## Related literature

For the biological activity of cyclo­hexenones, see: Hamon *et al.* (1996 ▶); Honda (2002 ▶); Keil *et al.* (1996 ▶). For green chemistry, see: Hoel & Nielsen (1999 ▶); Larhed *et al.* (1999 ▶). For ring puckering analysis, see: Cremer & Pople (1975 ▶). For stability of the temperature controller used in the data collection, see: Cosier & Glazer (1986 ▶).
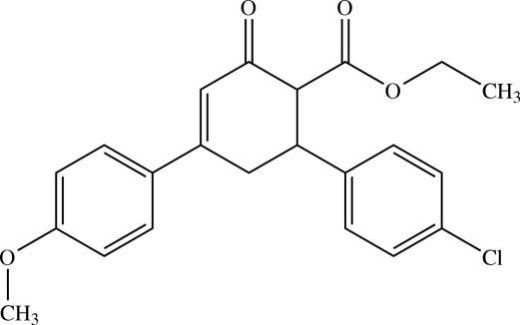


## Experimental

### 

#### Crystal data

C_22_H_21_ClO_4_
*M* *_r_* = 384.84
Monoclinic, 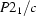

*a* = 11.9729 (3) Å
*b* = 8.1713 (2) Å
*c* = 39.2033 (8) Å
β = 98.990 (1)°
*V* = 3788.31 (15) Å^3^
*Z* = 8
Mo *K*α radiation
μ = 0.23 mm^−1^
*T* = 100 K
0.57 × 0.40 × 0.17 mm

#### Data collection

Bruker SMART APEXII CCD area-detector diffractometer
Absorption correction: multi-scan (*SADABS*; Bruker, 2005 ▶) *T*_min_ = 0.882, *T*_max_ = 0.962
60118 measured reflections
14422 independent reflections
10971 reflections with *I* > 2σ(*I*)
*R*_int_ = 0.043

#### Refinement

*R*[*F* ^2^ > 2σ(*F* ^2^)] = 0.072
*wR*(*F* ^2^) = 0.180
*S* = 1.16
14422 reflections
473 parameters
H-atom parameters constrained
Δρ_max_ = 1.03 e Å^−3^
Δρ_min_ = −0.96 e Å^−3^

### 

Data collection: *APEX2* (Bruker, 2005 ▶); cell refinement: *SAINT* (Bruker, 2005 ▶); data reduction: *SAINT*; program(s) used to solve structure: *SHELXTL* (Sheldrick, 2008 ▶); program(s) used to refine structure: *SHELXTL*; molecular graphics: *SHELXTL*; software used to prepare material for publication: *SHELXTL* and *PLATON* (Spek, 2009 ▶).

## Supplementary Material

Crystal structure: contains datablocks global, I. DOI: 10.1107/S1600536809016237/tk2440sup1.cif

Structure factors: contains datablocks I. DOI: 10.1107/S1600536809016237/tk2440Isup2.hkl

Additional supplementary materials:  crystallographic information; 3D view; checkCIF report

## Figures and Tables

**Table 1:** Hydrogen-bond geometry (Å, °)

*D*—H⋯*A*	*D*—H	H⋯*A*	*D*⋯*A*	*D*—H⋯*A*
C11*A*—H11*A*⋯O1*B*^i^	0.98	2.53	3.492 (2)	167
C11*B*—H11*B*⋯O1*A*^ii^	0.98	2.53	3.501 (2)	170
C12*A*—H12*B*⋯O2*B*	0.97	2.51	3.450 (2)	162
C12*B*—H12*C*⋯O2*A*^iii^	0.97	2.56	3.441 (2)	151
C15*B*—H15*B*⋯O4*B*^iv^	0.93	2.59	3.485 (3)	163
C20*B*—H20*D*⋯Cl1*A*^iv^	0.97	2.83	3.585 (2)	136
C22*A*—H22*A*⋯*Cg*1^ii^	0.97	2.83	3.666 (2)	146

Symmetry codes: (i) 
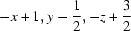
; (ii) 
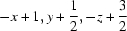
; (iii) 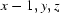; (iv) 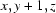.

**Fig. 1.**
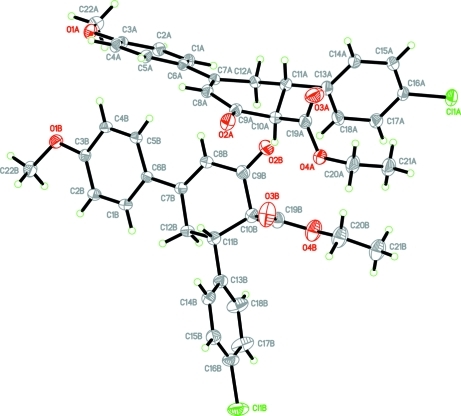
The molecular structure of (I), showing 50% probability displacement ellipsoids and the atom numbering scheme.

**Fig. 2.**
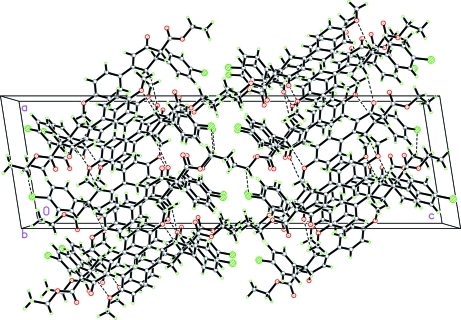
The crystal packing for (I), viewed along the b axis. Dashed lines indicate C—H···O and C—H···Cl contacts.

## supplementary crystallographic information

### Comment

Cyclohexenones are important intermediates in the synthesis of a wide variety of biologically active simple, condensed and bridged heterocycles. These cyclohexenones are also found to possess various types of biological activity, e.g. herbicidal (Keil *et al.*, 1996), in vitro inhibition of human platelet cylooxygenase (Hamon *et al.*, 1996), as an HMG-CoA reductase inhibitor (Honda, 2002), and displays anti-obesity properties (Honda, 2002). Today green chemistry plays an important role in chemical research. The large number of publications clearly indicates the development of this area of chemistry (Hoel & Nielsen, 1999; Larhed *et al.*, 1999).

The asymmetric unit of (I) (Fig. 1) comprises of two crystallographically independent molecules (A & B) with almost similar geometries. The cyclohex-3-ene unit in both the molecules adopt an envelope conformation with puckering parameters Q = 0.5107 (18) Å, θ = 124.6 (2)° and φ = 47.7 (2)° for molecule A, and Q = 0.50 (18) Å, θ = 56.0 (2)° and φ = 231.5 (2)° for molecule B (Cremer & Pople, 1975). The two benzene rings are inclined to each other forming dihedral angles of 66.82 (6)° (C1A—C6A:C13A—C18A) in molecule A and 73.68 (5)° (C1B—C6B: C13B—C18B) in molecule B.

Globally, the crystal packing comprises layers stabilized by C—H···O and C—H···Cl contacts (Fig. 2) together with C—H···π interactions (Table 1).

### Experimental

1-(*p*-Methoxyphenyl)-3-(*p*-chlorophenyl)-2-propene-1-one (0.01 mol), potassium carbonate (0.04 mol), ethyl acetoacetate (0.02 mol) were ground in a mortar using a pestle for uniform mixing. The paste formed was transferred to a 50 mL beaker and placed in a microwave oven operating at 160 W for 5 mins. The product (I) was poured into cold water, filtered, dried and recrystallized from ethanol-dioxane mixture; m. pt. = 411 - 412 K.

### Refinement

H atoms were positioned geometrically [C–H = 0.93–0.98 Å] and refined using a riding model with *U*_iso_(H) = 1.2*U*_eq_(C) and 1.5*U*_eq_(methyl C). A rotating–group model was used for the methyl groups. The maximum and minimum residual electron density peaks of 1.03 and -0.96 eÅ^-3^, respectively, were located 0.08 Å and 0.05 Å from the C20A and C21A  atoms, respectively

### Crystal data

**Table:**

C_22_H_21_ClO_4_	*F*(000) = 1616
*M**_r_* = 384.84	*D*_x_ = 1.349 Mg m^−^^3^
Monoclinic, *P*2_1_/*c*	Mo *K*α radiation, λ = 0.71073 Å
Hall symbol: -P 2ybc	Cell parameters from 9875 reflections
*a* = 11.9729 (3) Å	θ = 2.7–33.0°
*b* = 8.1713 (2) Å	µ = 0.23 mm^−^^1^
*c* = 39.2033 (8) Å	*T* = 100 K
β = 98.990 (1)°	Plate, colourless
*V* = 3788.31 (15) Å^3^	0.57 × 0.40 × 0.17 mm
*Z* = 8	

### Data collection

**Table:**

Bruker SMART APEXII CCD area-detector diffractometer	14422 independent reflections
Radiation source: fine-focus sealed tube	10971 reflections with *I* > 2σ(*I*)
graphite	*R*_int_ = 0.043
φ and ω scans	θ_max_ = 33.2°, θ_min_ = 2.6°
Absorption correction: multi-scan (*SADABS*; Bruker, 2005)	*h* = −17→18
*T*_min_ = 0.882, *T*_max_ = 0.962	*k* = −12→11
60118 measured reflections	*l* = −60→60

### Refinement

**Table:**

Refinement on *F*^2^	Primary atom site location: structure-invariant direct methods
Least-squares matrix: full	Secondary atom site location: difference Fourier map
*R*[*F*^2^ > 2σ(*F*^2^)] = 0.072	Hydrogen site location: inferred from neighbouring sites
*wR*(*F*^2^) = 0.180	H-atom parameters constrained
*S* = 1.16	*w* = 1/[σ^2^(*F*_o_^2^) + (0.064*P*)^2^ + 2.9459*P*] where *P* = (*F*_o_^2^ + 2*F*_c_^2^)/3
14422 reflections	(Δ/σ)_max_ < 0.002
473 parameters	Δρ_max_ = 1.03 e Å^−^^3^
0 restraints	Δρ_min_ = −0.96 e Å^−^^3^

### Special details

**Table:**

Experimental. The crystal was placed in the cold stream of an Oxford Cyrosystems Cobra open-flow nitrogen cryostat (Cosier & Glazer, 1986) operating at 100.0 (1) K.
Geometry. All e.s.d.'s (except the e.s.d. in the dihedral angle between two l.s. planes) are estimated using the full covariance matrix. The cell e.s.d.'s are taken into account individually in the estimation of e.s.d.'s in distances, angles and torsion angles; correlations between e.s.d.'s in cell parameters are only used when they are defined by crystal symmetry. An approximate (isotropic) treatment of cell e.s.d.'s is used for estimating e.s.d.'s involving l.s. planes.
Refinement. Refinement of *F*^2^ against ALL reflections. The weighted *R*-factor *wR* and goodness of fit *S* are based on *F*^2^, conventional *R*-factors *R* are based on *F*, with *F* set to zero for negative *F*^2^. The threshold expression of *F*^2^ > σ(*F*^2^) is used only for calculating *R*-factors(gt) *etc*. and is not relevant to the choice of reflections for refinement. *R*-factors based on *F*^2^ are statistically about twice as large as those based on *F*, and *R*- factors based on ALL data will be even larger.

### Fractional atomic coordinates and isotropic or equivalent isotropic displacement parameters (Å^2^)

**Table:**

	*x*	*y*	*z*	*U*_iso_*/*U*_eq_	
Cl1A	0.76330 (4)	0.14248 (6)	0.969549 (11)	0.02789 (11)	
O1A	0.45467 (10)	0.75170 (17)	0.66944 (3)	0.0223 (2)	
O2A	0.94698 (12)	1.01249 (18)	0.84718 (3)	0.0291 (3)	
O3A	1.04004 (14)	0.7270 (2)	0.89626 (4)	0.0409 (4)	
O4A	0.92250 (12)	0.85217 (18)	0.92726 (3)	0.0284 (3)	
C1A	0.56825 (14)	0.6458 (2)	0.75979 (4)	0.0203 (3)	
H1	0.5517	0.5806	0.7778	0.024*	
C2A	0.49553 (14)	0.6428 (2)	0.72817 (4)	0.0202 (3)	
H2	0.4320	0.5758	0.7252	0.024*	
C3A	0.51899 (13)	0.7407 (2)	0.70113 (4)	0.0179 (3)	
C4A	0.61617 (15)	0.8382 (2)	0.70575 (4)	0.0204 (3)	
H3	0.6326	0.9031	0.6877	0.024*	
C5A	0.68769 (15)	0.8387 (2)	0.73694 (4)	0.0199 (3)	
H4	0.7525	0.9031	0.7395	0.024*	
C6A	0.66492 (13)	0.7440 (2)	0.76511 (4)	0.0169 (3)	
C7A	0.73704 (14)	0.7533 (2)	0.79924 (4)	0.0177 (3)	
C8A	0.81593 (15)	0.8723 (2)	0.80671 (4)	0.0205 (3)	
H5	0.8307	0.9400	0.7889	0.025*	
C9A	0.87892 (15)	0.9002 (2)	0.84112 (4)	0.0220 (3)	
C10A	0.84923 (15)	0.7906 (2)	0.86985 (4)	0.0220 (3)	
H10A	0.7841	0.8370	0.8788	0.026*	
C11A	0.81864 (15)	0.6186 (2)	0.85548 (4)	0.0216 (3)	
H11A	0.8830	0.5787	0.8452	0.026*	
C12A	0.71788 (14)	0.6307 (2)	0.82654 (4)	0.0190 (3)	
H12A	0.7036	0.5240	0.8159	0.023*	
H12B	0.6513	0.6621	0.8363	0.023*	
C13A	0.80016 (15)	0.4985 (2)	0.88364 (4)	0.0218 (3)	
C14A	0.87730 (15)	0.3722 (2)	0.89192 (4)	0.0228 (3)	
H14A	0.9380	0.3619	0.8799	0.027*	
C15A	0.86513 (15)	0.2608 (2)	0.91800 (4)	0.0227 (3)	
H15A	0.9168	0.1760	0.9232	0.027*	
C16A	0.77543 (15)	0.2776 (2)	0.93598 (4)	0.0211 (3)	
C17A	0.69563 (16)	0.4008 (3)	0.92803 (5)	0.0268 (4)	
H17A	0.6348	0.4100	0.9400	0.032*	
C18A	0.70848 (17)	0.5101 (3)	0.90174 (5)	0.0274 (4)	
H18A	0.6552	0.5925	0.8961	0.033*	
C19A	0.94980 (17)	0.7852 (2)	0.89875 (5)	0.0250 (3)	
C20A	1.0027 (2)	0.8377 (3)	0.95884 (5)	0.0350 (3)	
H20A	1.0111	0.9422	0.9707	0.042*	
H20B	1.0760	0.8047	0.9536	0.042*	
C21A	0.9589 (2)	0.7117 (3)	0.98119 (5)	0.0350 (3)	
H21A	1.0087	0.7043	1.0028	0.052*	
H21B	0.9551	0.6074	0.9698	0.052*	
H21C	0.8847	0.7426	0.9853	0.052*	
C22A	0.35991 (15)	0.6440 (3)	0.66210 (5)	0.0261 (4)	
H22A	0.3232	0.6610	0.6388	0.039*	
H22B	0.3075	0.6660	0.6777	0.039*	
H22C	0.3853	0.5326	0.6648	0.039*	
Cl1B	0.23527 (5)	1.53507 (8)	0.970110 (14)	0.04101 (15)	
O1B	−0.03768 (10)	0.91313 (17)	0.67571 (3)	0.0228 (3)	
O2B	0.45811 (13)	0.65728 (19)	0.85379 (4)	0.0322 (3)	
O3B	0.55166 (14)	0.9617 (3)	0.89936 (5)	0.0459 (4)	
O4B	0.45395 (15)	0.8167 (2)	0.93271 (4)	0.0410 (3)	
C1B	0.07581 (14)	1.0158 (2)	0.76615 (4)	0.0208 (3)	
H6	0.0588	1.0796	0.7843	0.025*	
C2B	0.00365 (14)	1.0205 (2)	0.73455 (4)	0.0213 (3)	
H7	−0.0596	1.0881	0.7317	0.026*	
C3B	0.02715 (14)	0.9233 (2)	0.70740 (4)	0.0183 (3)	
C4B	0.12421 (14)	0.8254 (2)	0.71188 (4)	0.0200 (3)	
H8	0.1404	0.7607	0.6938	0.024*	
C5B	0.19597 (14)	0.8244 (2)	0.74301 (4)	0.0196 (3)	
H9	0.2610	0.7606	0.7454	0.023*	
C6B	0.17279 (13)	0.9183 (2)	0.77137 (4)	0.0171 (3)	
C7B	0.24481 (13)	0.9098 (2)	0.80548 (4)	0.0177 (3)	
C8B	0.32596 (15)	0.7943 (2)	0.81310 (4)	0.0211 (3)	
H10	0.3424	0.7277	0.7953	0.025*	
C9B	0.38899 (15)	0.7684 (2)	0.84758 (5)	0.0233 (3)	
C10B	0.36130 (15)	0.8793 (2)	0.87657 (4)	0.0230 (3)	
H10B	0.2999	0.8306	0.8870	0.028*	
C11B	0.32456 (15)	1.0481 (2)	0.86175 (4)	0.0224 (3)	
H11B	0.3873	1.0929	0.8514	0.027*	
C12B	0.22305 (14)	1.0301 (2)	0.83289 (4)	0.0199 (3)	
H12C	0.1579	0.9941	0.8428	0.024*	
H12D	0.2052	1.1361	0.8223	0.024*	
C13B	0.30004 (16)	1.1681 (2)	0.88921 (5)	0.0238 (3)	
C14B	0.36628 (16)	1.3074 (2)	0.89537 (4)	0.0249 (3)	
H14B	0.4254	1.3244	0.8830	0.030*	
C15B	0.34550 (17)	1.4219 (3)	0.91981 (5)	0.0277 (4)	
H15B	0.3898	1.5156	0.9236	0.033*	
C16B	0.25847 (17)	1.3951 (3)	0.93839 (5)	0.0284 (4)	
C17B	0.19055 (19)	1.2581 (3)	0.93277 (6)	0.0389 (5)	
H17B	0.1315	1.2420	0.9452	0.047*	
C18B	0.21188 (19)	1.1445 (3)	0.90817 (6)	0.0362 (5)	
H18B	0.1668	1.0515	0.9043	0.043*	
C19B	0.46667 (17)	0.8930 (3)	0.90359 (5)	0.0275 (4)	
C20B	0.5512 (2)	0.8290 (3)	0.96065 (6)	0.0410 (3)	
H20C	0.6171	0.7776	0.9537	0.049*	
H20D	0.5688	0.9429	0.9659	0.049*	
C21B	0.5210 (2)	0.7456 (3)	0.99117 (6)	0.0410 (3)	
H21D	0.5807	0.7601	1.0104	0.062*	
H21E	0.5104	0.6310	0.9863	0.062*	
H21F	0.4523	0.7914	0.9967	0.062*	
C22B	−0.12906 (15)	1.0268 (3)	0.66798 (5)	0.0269 (4)	
H22D	−0.1659	1.0104	0.6447	0.040*	
H22E	−0.1002	1.1364	0.6705	0.040*	
H22F	−0.1824	1.0097	0.6836	0.040*	

### Atomic displacement parameters (Å^2^)

**Table:**

	*U*^11^	*U*^22^	*U*^33^	*U*^12^	*U*^13^	*U*^23^
Cl1A	0.0339 (2)	0.0278 (2)	0.02181 (18)	−0.00302 (18)	0.00375 (15)	0.00647 (16)
O1A	0.0201 (6)	0.0265 (7)	0.0197 (5)	−0.0030 (5)	0.0011 (4)	0.0015 (5)
O2A	0.0337 (7)	0.0281 (7)	0.0243 (6)	−0.0102 (6)	0.0009 (5)	−0.0009 (5)
O3A	0.0383 (9)	0.0536 (11)	0.0294 (7)	0.0118 (8)	0.0007 (6)	−0.0097 (7)
O4A	0.0331 (7)	0.0305 (8)	0.0202 (6)	0.0055 (6)	−0.0002 (5)	0.0003 (5)
C1A	0.0223 (7)	0.0186 (8)	0.0208 (7)	−0.0019 (6)	0.0058 (6)	0.0031 (6)
C2A	0.0188 (7)	0.0208 (8)	0.0218 (7)	−0.0027 (6)	0.0053 (5)	0.0007 (6)
C3A	0.0181 (7)	0.0171 (7)	0.0189 (7)	0.0018 (6)	0.0042 (5)	−0.0003 (5)
C4A	0.0238 (8)	0.0202 (8)	0.0176 (7)	−0.0035 (6)	0.0047 (5)	0.0017 (6)
C5A	0.0221 (7)	0.0196 (8)	0.0187 (7)	−0.0041 (6)	0.0047 (5)	−0.0004 (6)
C6A	0.0187 (7)	0.0151 (7)	0.0175 (6)	0.0002 (5)	0.0047 (5)	−0.0001 (5)
C7A	0.0194 (7)	0.0167 (7)	0.0175 (6)	0.0029 (6)	0.0047 (5)	0.0008 (5)
C8A	0.0246 (8)	0.0191 (8)	0.0182 (7)	−0.0012 (6)	0.0041 (6)	0.0015 (6)
C9A	0.0263 (8)	0.0206 (8)	0.0188 (7)	−0.0007 (6)	0.0030 (6)	−0.0007 (6)
C10A	0.0254 (8)	0.0204 (8)	0.0202 (7)	0.0028 (6)	0.0041 (6)	−0.0007 (6)
C11A	0.0235 (8)	0.0229 (9)	0.0187 (7)	0.0020 (6)	0.0040 (6)	0.0011 (6)
C12A	0.0210 (7)	0.0171 (8)	0.0189 (7)	0.0011 (6)	0.0035 (5)	0.0017 (6)
C13A	0.0236 (8)	0.0215 (8)	0.0193 (7)	−0.0003 (6)	0.0005 (6)	0.0026 (6)
C14A	0.0230 (8)	0.0245 (9)	0.0209 (7)	0.0004 (6)	0.0032 (6)	0.0017 (6)
C15A	0.0225 (8)	0.0221 (9)	0.0228 (7)	0.0031 (6)	0.0015 (6)	0.0026 (6)
C16A	0.0251 (8)	0.0211 (8)	0.0166 (6)	−0.0017 (6)	0.0013 (6)	0.0019 (6)
C17A	0.0267 (9)	0.0311 (10)	0.0237 (8)	0.0059 (7)	0.0076 (6)	0.0046 (7)
C18A	0.0279 (9)	0.0282 (10)	0.0264 (8)	0.0088 (7)	0.0054 (7)	0.0076 (7)
C19A	0.0312 (9)	0.0228 (9)	0.0199 (7)	0.0003 (7)	0.0009 (6)	0.0000 (6)
C20A	0.0417 (8)	0.0371 (9)	0.0238 (6)	−0.0014 (7)	−0.0021 (5)	0.0038 (6)
C21A	0.0417 (8)	0.0371 (9)	0.0238 (6)	−0.0014 (7)	−0.0021 (5)	0.0038 (6)
C22A	0.0185 (7)	0.0328 (10)	0.0266 (8)	−0.0043 (7)	0.0025 (6)	−0.0035 (7)
Cl1B	0.0409 (3)	0.0450 (3)	0.0356 (3)	0.0091 (2)	0.0014 (2)	−0.0205 (2)
O1B	0.0202 (6)	0.0274 (7)	0.0204 (5)	0.0026 (5)	0.0024 (4)	0.0002 (5)
O2B	0.0327 (7)	0.0336 (8)	0.0290 (7)	0.0127 (6)	0.0011 (5)	0.0021 (6)
O3B	0.0327 (8)	0.0601 (12)	0.0425 (9)	−0.0162 (8)	−0.0013 (7)	0.0143 (8)
O4B	0.0416 (6)	0.0488 (7)	0.0299 (5)	−0.0111 (5)	−0.0030 (4)	0.0040 (5)
C1B	0.0216 (7)	0.0197 (8)	0.0218 (7)	0.0028 (6)	0.0058 (6)	−0.0030 (6)
C2B	0.0187 (7)	0.0230 (8)	0.0228 (7)	0.0044 (6)	0.0055 (6)	−0.0004 (6)
C3B	0.0190 (7)	0.0179 (8)	0.0187 (7)	−0.0015 (6)	0.0054 (5)	0.0013 (5)
C4B	0.0217 (7)	0.0197 (8)	0.0197 (7)	0.0027 (6)	0.0068 (5)	−0.0009 (6)
C5B	0.0212 (7)	0.0182 (8)	0.0202 (7)	0.0034 (6)	0.0059 (5)	0.0004 (6)
C6B	0.0182 (7)	0.0150 (7)	0.0187 (6)	0.0006 (5)	0.0049 (5)	−0.0002 (5)
C7B	0.0175 (7)	0.0163 (7)	0.0203 (7)	−0.0020 (5)	0.0056 (5)	−0.0009 (5)
C8B	0.0220 (7)	0.0203 (8)	0.0215 (7)	0.0021 (6)	0.0044 (6)	−0.0007 (6)
C9B	0.0217 (8)	0.0230 (9)	0.0252 (8)	0.0004 (6)	0.0035 (6)	0.0007 (6)
C10B	0.0239 (8)	0.0230 (9)	0.0222 (7)	−0.0036 (6)	0.0036 (6)	0.0003 (6)
C11B	0.0228 (8)	0.0219 (8)	0.0226 (7)	−0.0016 (6)	0.0045 (6)	−0.0010 (6)
C12B	0.0199 (7)	0.0194 (8)	0.0206 (7)	0.0003 (6)	0.0038 (5)	−0.0036 (6)
C13B	0.0247 (8)	0.0241 (9)	0.0220 (7)	−0.0011 (7)	0.0015 (6)	−0.0041 (6)
C14B	0.0267 (8)	0.0275 (9)	0.0201 (7)	−0.0011 (7)	0.0022 (6)	−0.0012 (6)
C15B	0.0313 (9)	0.0242 (9)	0.0258 (8)	−0.0026 (7)	−0.0007 (7)	−0.0028 (7)
C16B	0.0277 (9)	0.0316 (10)	0.0243 (8)	0.0048 (7)	−0.0005 (7)	−0.0104 (7)
C17B	0.0301 (10)	0.0517 (15)	0.0371 (11)	−0.0113 (10)	0.0124 (8)	−0.0192 (10)
C18B	0.0352 (10)	0.0386 (12)	0.0375 (10)	−0.0153 (9)	0.0137 (8)	−0.0166 (9)
C19B	0.0285 (9)	0.0288 (10)	0.0238 (8)	−0.0024 (7)	−0.0002 (7)	0.0012 (7)
C20B	0.0416 (6)	0.0488 (7)	0.0299 (5)	−0.0111 (5)	−0.0030 (4)	0.0040 (5)
C21B	0.0416 (6)	0.0488 (7)	0.0299 (5)	−0.0111 (5)	−0.0030 (4)	0.0040 (5)
C22B	0.0196 (8)	0.0363 (11)	0.0250 (8)	0.0041 (7)	0.0041 (6)	0.0058 (7)

### Geometric parameters (Å, °)

**Table:**

Cl1A—C16A	1.7412 (18)	Cl1B—C16B	1.7435 (19)
O1A—C3A	1.358 (2)	O1B—C3B	1.361 (2)
O1A—C22A	1.429 (2)	O1B—C22B	1.431 (2)
O2A—C9A	1.226 (2)	O2B—C9B	1.227 (2)
O3A—C19A	1.198 (2)	O3B—C19B	1.196 (3)
O4A—C19A	1.330 (2)	O4B—C19B	1.331 (2)
O4A—C20A	1.448 (2)	O4B—C20B	1.472 (3)
C1A—C6A	1.397 (2)	C1B—C2B	1.396 (2)
C1A—C2A	1.399 (2)	C1B—C6B	1.397 (2)
C1A—H1	0.9300	C1B—H6	0.9300
C2A—C3A	1.391 (2)	C2B—C3B	1.392 (2)
C2A—H2	0.9300	C2B—H7	0.9300
C3A—C4A	1.398 (2)	C3B—C4B	1.399 (2)
C4A—C5A	1.379 (2)	C4B—C5B	1.379 (2)
C4A—H3	0.9300	C4B—H8	0.9300
C5A—C6A	1.410 (2)	C5B—C6B	1.413 (2)
C5A—H4	0.9300	C5B—H9	0.9300
C6A—C7A	1.477 (2)	C6B—C7B	1.475 (2)
C7A—C8A	1.355 (2)	C7B—C8B	1.354 (2)
C7A—C12A	1.510 (2)	C7B—C12B	1.509 (2)
C8A—C9A	1.457 (2)	C8B—C9B	1.457 (2)
C8A—H5	0.9300	C8B—H10	0.9300
C9A—C10A	1.524 (2)	C9B—C10B	1.529 (3)
C10A—C19A	1.520 (3)	C10B—C19B	1.519 (3)
C10A—C11A	1.537 (3)	C10B—C11B	1.534 (3)
C10A—H10A	0.9800	C10B—H10B	0.9800
C11A—C13A	1.519 (2)	C11B—C13B	1.518 (3)
C11A—C12A	1.525 (2)	C11B—C12B	1.533 (2)
C11A—H11A	0.9800	C11B—H11B	0.9800
C12A—H12A	0.9700	C12B—H12C	0.9700
C12A—H12B	0.9700	C12B—H12D	0.9700
C13A—C14A	1.389 (3)	C13B—C14B	1.387 (3)
C13A—C18A	1.400 (3)	C13B—C18B	1.396 (3)
C14A—C15A	1.394 (2)	C14B—C15B	1.389 (3)
C14A—H14A	0.9300	C14B—H14B	0.9300
C15A—C16A	1.380 (2)	C15B—C16B	1.379 (3)
C15A—H15A	0.9300	C15B—H15B	0.9300
C16A—C17A	1.389 (3)	C16B—C17B	1.381 (3)
C17A—C18A	1.390 (3)	C17B—C18B	1.391 (3)
C17A—H17A	0.9300	C17B—H17B	0.9300
C18A—H18A	0.9300	C18B—H18B	0.9300
C20A—C21A	1.499 (3)	C20B—C21B	1.470 (3)
C20A—H20A	0.9700	C20B—H20C	0.9700
C20A—H20B	0.9700	C20B—H20D	0.9700
C21A—H21A	0.9600	C21B—H21D	0.9600
C21A—H21B	0.9600	C21B—H21E	0.9600
C21A—H21C	0.9600	C21B—H21F	0.9600
C22A—H22A	0.9600	C22B—H22D	0.9600
C22A—H22B	0.9600	C22B—H22E	0.9600
C22A—H22C	0.9600	C22B—H22F	0.9600
			
C3A—O1A—C22A	117.53 (14)	C3B—O1B—C22B	117.52 (14)
C19A—O4A—C20A	117.88 (16)	C19B—O4B—C20B	115.04 (17)
C6A—C1A—C2A	121.93 (15)	C2B—C1B—C6B	122.08 (15)
C6A—C1A—H1	119.0	C2B—C1B—H6	119.0
C2A—C1A—H1	119.0	C6B—C1B—H6	119.0
C3A—C2A—C1A	119.54 (16)	C3B—C2B—C1B	119.46 (16)
C3A—C2A—H2	120.2	C3B—C2B—H7	120.3
C1A—C2A—H2	120.2	C1B—C2B—H7	120.3
O1A—C3A—C2A	125.56 (15)	O1B—C3B—C2B	125.33 (15)
O1A—C3A—C4A	114.96 (14)	O1B—C3B—C4B	115.09 (15)
C2A—C3A—C4A	119.47 (15)	C2B—C3B—C4B	119.58 (15)
C5A—C4A—C3A	120.34 (15)	C5B—C4B—C3B	120.34 (15)
C5A—C4A—H3	119.8	C5B—C4B—H8	119.8
C3A—C4A—H3	119.8	C3B—C4B—H8	119.8
C4A—C5A—C6A	121.60 (16)	C4B—C5B—C6B	121.43 (15)
C4A—C5A—H4	119.2	C4B—C5B—H9	119.3
C6A—C5A—H4	119.2	C6B—C5B—H9	119.3
C1A—C6A—C5A	117.10 (15)	C1B—C6B—C5B	117.08 (15)
C1A—C6A—C7A	121.23 (14)	C1B—C6B—C7B	120.96 (14)
C5A—C6A—C7A	121.62 (15)	C5B—C6B—C7B	121.92 (15)
C8A—C7A—C6A	121.44 (15)	C8B—C7B—C6B	122.04 (15)
C8A—C7A—C12A	120.09 (15)	C8B—C7B—C12B	119.66 (15)
C6A—C7A—C12A	118.42 (14)	C6B—C7B—C12B	118.25 (14)
C7A—C8A—C9A	123.66 (15)	C7B—C8B—C9B	123.75 (16)
C7A—C8A—H5	118.2	C7B—C8B—H10	118.1
C9A—C8A—H5	118.2	C9B—C8B—H10	118.1
O2A—C9A—C8A	121.92 (16)	O2B—C9B—C8B	121.78 (17)
O2A—C9A—C10A	121.42 (15)	O2B—C9B—C10B	120.58 (16)
C8A—C9A—C10A	116.49 (15)	C8B—C9B—C10B	117.54 (16)
C19A—C10A—C9A	108.61 (15)	C19B—C10B—C9B	107.99 (15)
C19A—C10A—C11A	111.23 (15)	C19B—C10B—C11B	110.80 (15)
C9A—C10A—C11A	109.63 (14)	C9B—C10B—C11B	109.50 (14)
C19A—C10A—H10A	109.1	C19B—C10B—H10B	109.5
C9A—C10A—H10A	109.1	C9B—C10B—H10B	109.5
C11A—C10A—H10A	109.1	C11B—C10B—H10B	109.5
C13A—C11A—C12A	113.23 (15)	C13B—C11B—C12B	111.51 (15)
C13A—C11A—C10A	112.21 (14)	C13B—C11B—C10B	112.82 (15)
C12A—C11A—C10A	109.22 (14)	C12B—C11B—C10B	109.90 (15)
C13A—C11A—H11A	107.3	C13B—C11B—H11B	107.5
C12A—C11A—H11A	107.3	C12B—C11B—H11B	107.5
C10A—C11A—H11A	107.3	C10B—C11B—H11B	107.5
C7A—C12A—C11A	112.34 (14)	C7B—C12B—C11B	112.52 (14)
C7A—C12A—H12A	109.1	C7B—C12B—H12C	109.1
C11A—C12A—H12A	109.1	C11B—C12B—H12C	109.1
C7A—C12A—H12B	109.1	C7B—C12B—H12D	109.1
C11A—C12A—H12B	109.1	C11B—C12B—H12D	109.1
H12A—C12A—H12B	107.9	H12C—C12B—H12D	107.8
C14A—C13A—C18A	118.50 (16)	C14B—C13B—C18B	118.69 (17)
C14A—C13A—C11A	118.87 (16)	C14B—C13B—C11B	119.00 (16)
C18A—C13A—C11A	122.63 (16)	C18B—C13B—C11B	122.30 (17)
C13A—C14A—C15A	121.00 (17)	C13B—C14B—C15B	120.91 (18)
C13A—C14A—H14A	119.5	C13B—C14B—H14B	119.5
C15A—C14A—H14A	119.5	C15B—C14B—H14B	119.5
C16A—C15A—C14A	119.22 (17)	C16B—C15B—C14B	119.28 (19)
C16A—C15A—H15A	120.4	C16B—C15B—H15B	120.4
C14A—C15A—H15A	120.4	C14B—C15B—H15B	120.4
C15A—C16A—C17A	121.35 (16)	C15B—C16B—C17B	121.28 (18)
C15A—C16A—Cl1A	119.08 (14)	C15B—C16B—Cl1B	119.37 (16)
C17A—C16A—Cl1A	119.57 (14)	C17B—C16B—Cl1B	119.35 (16)
C16A—C17A—C18A	118.69 (17)	C16B—C17B—C18B	119.0 (2)
C16A—C17A—H17A	120.7	C16B—C17B—H17B	120.5
C18A—C17A—H17A	120.7	C18B—C17B—H17B	120.5
C17A—C18A—C13A	121.22 (17)	C17B—C18B—C13B	120.9 (2)
C17A—C18A—H18A	119.4	C17B—C18B—H18B	119.6
C13A—C18A—H18A	119.4	C13B—C18B—H18B	119.6
O3A—C19A—O4A	125.08 (17)	O3B—C19B—O4B	123.55 (18)
O3A—C19A—C10A	124.77 (17)	O3B—C19B—C10B	124.74 (18)
O4A—C19A—C10A	110.14 (16)	O4B—C19B—C10B	111.70 (17)
O4A—C20A—C21A	108.05 (18)	C21B—C20B—O4B	107.91 (19)
O4A—C20A—H20A	110.1	C21B—C20B—H20C	110.1
C21A—C20A—H20A	110.1	O4B—C20B—H20C	110.1
O4A—C20A—H20B	110.1	C21B—C20B—H20D	110.1
C21A—C20A—H20B	110.1	O4B—C20B—H20D	110.1
H20A—C20A—H20B	108.4	H20C—C20B—H20D	108.4
C20A—C21A—H21A	109.5	C20B—C21B—H21D	109.5
C20A—C21A—H21B	109.5	C20B—C21B—H21E	109.5
H21A—C21A—H21B	109.5	H21D—C21B—H21E	109.5
C20A—C21A—H21C	109.5	C20B—C21B—H21F	109.5
H21A—C21A—H21C	109.5	H21D—C21B—H21F	109.5
H21B—C21A—H21C	109.5	H21E—C21B—H21F	109.5
O1A—C22A—H22A	109.5	O1B—C22B—H22D	109.5
O1A—C22A—H22B	109.5	O1B—C22B—H22E	109.5
H22A—C22A—H22B	109.5	H22D—C22B—H22E	109.5
O1A—C22A—H22C	109.5	O1B—C22B—H22F	109.5
H22A—C22A—H22C	109.5	H22D—C22B—H22F	109.5
H22B—C22A—H22C	109.5	H22E—C22B—H22F	109.5
			
C6A—C1A—C2A—C3A	−0.4 (3)	C6B—C1B—C2B—C3B	1.2 (3)
C22A—O1A—C3A—C2A	−5.5 (2)	C22B—O1B—C3B—C2B	8.7 (2)
C22A—O1A—C3A—C4A	174.43 (15)	C22B—O1B—C3B—C4B	−171.47 (15)
C1A—C2A—C3A—O1A	−178.75 (16)	C1B—C2B—C3B—O1B	178.15 (16)
C1A—C2A—C3A—C4A	1.3 (3)	C1B—C2B—C3B—C4B	−1.6 (3)
O1A—C3A—C4A—C5A	179.41 (16)	O1B—C3B—C4B—C5B	−179.46 (16)
C2A—C3A—C4A—C5A	−0.6 (3)	C2B—C3B—C4B—C5B	0.4 (3)
C3A—C4A—C5A—C6A	−0.9 (3)	C3B—C4B—C5B—C6B	1.4 (3)
C2A—C1A—C6A—C5A	−1.0 (3)	C2B—C1B—C6B—C5B	0.5 (3)
C2A—C1A—C6A—C7A	176.27 (16)	C2B—C1B—C6B—C7B	−177.18 (16)
C4A—C5A—C6A—C1A	1.7 (3)	C4B—C5B—C6B—C1B	−1.8 (3)
C4A—C5A—C6A—C7A	−175.58 (16)	C4B—C5B—C6B—C7B	175.83 (16)
C1A—C6A—C7A—C8A	−165.71 (16)	C1B—C6B—C7B—C8B	167.12 (17)
C5A—C6A—C7A—C8A	11.5 (2)	C5B—C6B—C7B—C8B	−10.5 (3)
C1A—C6A—C7A—C12A	11.8 (2)	C1B—C6B—C7B—C12B	−10.4 (2)
C5A—C6A—C7A—C12A	−171.04 (15)	C5B—C6B—C7B—C12B	172.03 (15)
C6A—C7A—C8A—C9A	171.75 (16)	C6B—C7B—C8B—C9B	−171.91 (16)
C12A—C7A—C8A—C9A	−5.7 (3)	C12B—C7B—C8B—C9B	5.5 (3)
C7A—C8A—C9A—O2A	−177.50 (18)	C7B—C8B—C9B—O2B	176.40 (18)
C7A—C8A—C9A—C10A	−2.1 (3)	C7B—C8B—C9B—C10B	−0.2 (3)
O2A—C9A—C10A—C19A	−28.0 (2)	O2B—C9B—C10B—C19B	31.1 (2)
C8A—C9A—C10A—C19A	156.65 (16)	C8B—C9B—C10B—C19B	−152.28 (17)
O2A—C9A—C10A—C11A	−149.68 (18)	O2B—C9B—C10B—C11B	151.80 (18)
C8A—C9A—C10A—C11A	34.9 (2)	C8B—C9B—C10B—C11B	−31.5 (2)
C19A—C10A—C11A—C13A	53.8 (2)	C19B—C10B—C11B—C13B	−58.7 (2)
C9A—C10A—C11A—C13A	173.98 (15)	C9B—C10B—C11B—C13B	−177.67 (15)
C19A—C10A—C11A—C12A	−179.76 (14)	C19B—C10B—C11B—C12B	176.23 (14)
C9A—C10A—C11A—C12A	−59.61 (18)	C9B—C10B—C11B—C12B	57.21 (18)
C8A—C7A—C12A—C11A	−20.7 (2)	C8B—C7B—C12B—C11B	21.9 (2)
C6A—C7A—C12A—C11A	161.77 (14)	C6B—C7B—C12B—C11B	−160.50 (15)
C13A—C11A—C12A—C7A	178.76 (14)	C13B—C11B—C12B—C7B	−179.27 (15)
C10A—C11A—C12A—C7A	52.94 (18)	C10B—C11B—C12B—C7B	−53.39 (19)
C12A—C11A—C13A—C14A	124.12 (18)	C12B—C11B—C13B—C14B	−119.08 (18)
C10A—C11A—C13A—C14A	−111.67 (19)	C10B—C11B—C13B—C14B	116.67 (19)
C12A—C11A—C13A—C18A	−56.1 (2)	C12B—C11B—C13B—C18B	59.9 (2)
C10A—C11A—C13A—C18A	68.1 (2)	C10B—C11B—C13B—C18B	−64.3 (2)
C18A—C13A—C14A—C15A	−1.0 (3)	C18B—C13B—C14B—C15B	−0.2 (3)
C11A—C13A—C14A—C15A	178.80 (16)	C11B—C13B—C14B—C15B	178.82 (17)
C13A—C14A—C15A—C16A	−0.6 (3)	C13B—C14B—C15B—C16B	0.7 (3)
C14A—C15A—C16A—C17A	1.6 (3)	C14B—C15B—C16B—C17B	−1.0 (3)
C14A—C15A—C16A—Cl1A	−177.76 (14)	C14B—C15B—C16B—Cl1B	177.82 (15)
C15A—C16A—C17A—C18A	−1.1 (3)	C15B—C16B—C17B—C18B	0.9 (4)
Cl1A—C16A—C17A—C18A	178.31 (16)	Cl1B—C16B—C17B—C18B	−178.01 (19)
C16A—C17A—C18A—C13A	−0.5 (3)	C16B—C17B—C18B—C13B	−0.3 (4)
C14A—C13A—C18A—C17A	1.5 (3)	C14B—C13B—C18B—C17B	0.0 (3)
C11A—C13A—C18A—C17A	−178.23 (18)	C11B—C13B—C18B—C17B	−179.0 (2)
C20A—O4A—C19A—O3A	−7.5 (3)	C20B—O4B—C19B—O3B	2.9 (3)
C20A—O4A—C19A—C10A	171.64 (17)	C20B—O4B—C19B—C10B	−178.08 (19)
C9A—C10A—C19A—O3A	−65.2 (3)	C9B—C10B—C19B—O3B	68.3 (3)
C11A—C10A—C19A—O3A	55.6 (3)	C11B—C10B—C19B—O3B	−51.6 (3)
C9A—C10A—C19A—O4A	115.70 (17)	C9B—C10B—C19B—O4B	−110.75 (19)
C11A—C10A—C19A—O4A	−123.55 (17)	C11B—C10B—C19B—O4B	129.32 (18)
C19A—O4A—C20A—C21A	−105.0 (2)	C19B—O4B—C20B—C21B	177.3 (2)

### Hydrogen-bond geometry (Å, °)

**Table:**

*D*—H···*A*	*D*—H	H···*A*	*D*···*A*	*D*—H···*A*
C11A—H11A···O1B^i^	0.98	2.53	3.492 (2)	167
C11B—H11B···O1A^ii^	0.98	2.53	3.501 (2)	170
C12A—H12B···O2B	0.97	2.51	3.450 (2)	162
C12B—H12C···O2A^iii^	0.97	2.56	3.441 (2)	151
C15B—H15B···O4B^iv^	0.93	2.59	3.485 (3)	163
C20B—H20D···Cl1A^iv^	0.97	2.83	3.585 (2)	136
C22A—H22A···Cg1^ii^	0.97	2.83	3.666 (2)	146

Symmetry codes: (i) −*x*+1, *y*−1/2, −*z*+3/2; (ii) −*x*+1, *y*+1/2, −*z*+3/2; (iii) *x*−1, *y*, *z*; (iv) *x*, *y*+1, *z*.

## References

[bb1] Bruker (2005). *APEX2*, *SAINT* and *SADABS* Bruker AXS Inc., Madison, Wisconsin, USA.

[bb2] Cosier, J. & Glazer, A. M. (1986). *J. Appl. Cryst.* **19**, 105–107.

[bb3] Cremer, D. & Pople, J. A. (1975). *J. Am. Chem. Soc.* **97**, 1354–1358.

[bb4] Hamon, D. P. G., Hayball, P. J., Massy-Westropp, R. A., Newton, J. L. & Tamblyn, J. G. (1996). *Tetrahedron Asymmetry*, **7**, 263–272.

[bb5] Hoel, A. M. L. & Nielsen, J. (1999). *Tetrahedron Lett.* **40**, 3941–3944.

[bb6] Honda. (2002). *J. Synth. Org. Chem. Jpn*, **60**, 1104–1111.

[bb7] Keil, M., Schirmer, U., Kolassa, D., Kast, J., Wuerzer, B. & Meyer, N. (1996). US Patent No. 5 554 582.

[bb8] Larhed, M., Lindeberg, G. & Hallberg, A. (1999). *Tetrahedron Lett.*, **37**, 8219–8222.

[bb9] Sheldrick, G. M. (2008). *Acta Cryst.* A**64**, 112–122. 10.1107/S0108767307043930 18156677

[bb10] Spek, A. L. (2009). *Acta Cryst.* D**65**, 148–155. 10.1107/S090744490804362X PMC2631630 19171970

